# 

**Published:** 1923-01

**Authors:** 


### Bristol Medico-Chirurgical Society.

### PAST PRESIDENTS.

1874—75. FREDERICK BRITTAN, M.D., B.A. Dub.
1875—76. ROBERT WILLIAM COE.
1876—77. SAMUEL MARTYN, M.D. Ed.
1877—78. AUGUSTIN PRICHARD, M.D. Berlin.
1878—79. HENRY EDWARD FRIPP, M.D. Würzburg.
1879—80. WILLIAM MICHELL CLARKE.
1880—81. JOSEPH GRIFFITHS SWAYNE, M.D. Lond.
1881—82. EDWARD LONG FOX, M.D. Oxon.
1882—83. JAMES GEORGE DAVEY, M.D. St. And.
1883—84. WILLIAM JOHNSTONE FYFFE, M.D., B.A. Dub.
1884—85. GEORGE FORSTER BURDER, M.D. Aberd.
1885—86. WILLIAM HENRY SPENCER, M.D., M.A. Cantab.
1886—87. FRANCIS POOLE LANSDOWN.
1887—88. LEMUEL MATTHEWS GRIFFITHS.
1888—89. ROBERT SHINGLETON SMITH, M.D., B.Sc. Lond.
1889—90. NELSON CONGREVE DOBSON, Ch.M. Bristol.
1890—91. SAMUEL HENRY SWAYNE.
1891—92. FRANCIS RICHARDSON CROSS, M.B.Lond., LL D.Bristol.
1892—93. EDWARD MARKHAM SKERRITT, M D., B.S., B.A. Lond.
1893—94. JAMES GREIG SMITH, M.B., C.M., M.A. Aberd., F.R.S.E.
1894—95. ALFRED JAMES HARRISON, M.B. Lond.
1895—96. ARTHUR WILLIAM PRICHARD.
1896—97. ALFRED EDWARD AUST LAWRENCE, M.D., C.M. Aberd.
1897—98. JOHN EDWARD SHAW, M.B., C.M. Ed.
1898—99. ROBERT ROXBURGH, M.B. Ed.
1899—00. WILLIAM HENRY HARSANT.
1900—01. DAVID SAMUEL DAVIES, M.D.,Lond.
1901—02. BARCLAY JOSIAH BARON, M.B., C.M. Ed.
1902—03. GEORGE MUNRO SMITH, M.D. Bristol.
1903—04. JAMES PAUL BUSH, C.M.G., C.B.E., Ch.M. Bristol.
1904—05. REGINALD EAGER, M.D. Lond.
1905—06. JOHN DACRE.
1906—07. JAMES TAYLOR.
1907—08. HENRY WALDO, M.D., C.M. Aberd.
1908—09. JOHN MICHELL CLARKE, M.D , M.A. Cantab.
1909—10. JAMES SWAIN, C.B., C.B.E., M.D., M.S. Lond.
1910—11. BERTRAM MITFORD HERON ROGERS, M.D., B.Ch., B.A. Oxon.
1911—12. CHARLES ALEXANDER MORTON.
1912—13. WALTER CARLESS SWAYNE, M.D., B.S. Lond. and Bristol.
1913—14. PATRICK WATSON-WILLIAMS, M.D. Lond.
1914—15. WILLIAM HARRY CHRISTOPHER NEWNHAM, M.A., M B. Cantab.
1915—16 GEORGE PARKER, M.A., M.D. Cantab.
1916—17 FRANCIS HENRY EDGEWORTH, M.A., M.D. Cantab., D.Sc. Lond.
1917—18 FREDERICK LACE.
1918—19 ROBERT GUTHRIE POOLE LANSDOWN, M.D., B.S. Durh.
1919—20 I. WALKER HALL, M.D., Ch.B. Vict.
1920—21 LEOPOLD ERNEST VALENTINE EVERY-CLAYTON, M.D. Lond.
1921—22 CYRIL HUTCHINSON WALKER, M.B., B.A. Cantab.

1923.

### LIST OF SUBSCRIBERS

TO THE

### Bristol Medico-Chirurgical Journal.

### HONORARY MEMBERS OF THE SOCIETY.

| | |
|---|---|
| GRIFFITHS, L. M. ... ... ... ... | 11 Pembroke Road, Clifton. |
| OWEN, SIR ISAMBARD, D.C.L., M.D. | Abbey Gate, Beach Road, Weston-super-Mare. |

### SUBSCRIBERS.

*Those marked * are Members of the Society.*

| | |
|---|---|
| ACKLAND, J. McKNO ... ... ... ... ... ... | 1 Barnfield Crescent, Exeter. |
| *ACKLAND, W. R., M.D.S. Brist. ... ... ... | 5 Rodney Place, Clifton. |
| *ADAMS, A. W., M.B., B.Sc. Lond. ... ... ... | 9 Mortimer Road, Clifton. |
| ADYE, W. J. A. ... ... ... .. ... ... ... | St. Margarets, Bradford-on Avon, Wilts. |
| *ALEXANDER, A., M.A. ... ... ... ... ... .. | 23 Manilla Road, Clifton. |
| *ALEXANDER, D. A., M.B., Ch.B. Vict. ... ... | 30 Berkeley Square, Clifton. |
| *ASKINS, R. A., M.D., B.Ch., B.A.O. Dubl.... | Guildhall, Bristol. |
| *AUBREY, T., M.B. Lond. ... ... ... ... ... | Bitton, near Bristol. |
| *BAKER, LILY A., M.D., B.Ch., B.A.O., R.U.I. ... | 21 Victoria Square, Clifton. |
| *BALL, E. J., M.A., Ph.D., Heidl. ... ... ... | 120 Pembroke Road, Bristol. |

### LIST OF SUBSCRIBERS.

| | |
|---|---|
| *Barber, M. C., M.B., Ch.B. Brist. ... ... ... | Ingledene, High Street Staple Hill, Bristol. |
| *Barker, G. H., M.D., B.Sc. Lond. ... ... ... | 124 Redland Road, Bristol. |
| *Bartholomew, E. U. ... ... ... ... ... ... | 7 Clifton Hill, Clifton. |
| *Bergin, F. G. ... ... ... ... ... ... ... ... ... | 31 Oakfield Road, Clifton. |
| Bernard, C. ... ... ... ... ... ... ... ... ... | 704 Fishponds Road, Fishponds, Bristol. |
| Berry, F. C., M.D., B.Ch., B.A. Dub. ... ... | Locarno, Burnham, Somerset. |
| *Birrell, J. A., M.B., B.S. Lond. ... ... | 28 Berkeley Square, Clifton. |
| *Blacker, A. E.... ... ... ... ... ... ... ... | 20 Victoria Square, Clifton. |
| Blackett, J. F., M.D., B.S. Lond. ... ... ... | Hamsterley, Bloomfield Park, Bath. |
| *Blagg, Arthur F., M.D. Durh.... ... ... ... | 28 Caledonia Place, Clifton. |
| *Bodman, C. O., M.D. Durh. ... ... ... ... | 17 Upper Belgrave Road, Clifton. |
| *Bodman, J. Hervey, M.D. Lond. ... ... ... | 9 Whiteladies Road, Clifton. |
| *Bowker, G. E., M.D. Edin.... ... ... ... ... | 11 North Parade, Bath. |
| *Bradley, E. J., M.B. ... ... ... ... ... ... | 21 Foregate Street, Stafford. |
| *Bray, G., Lt.-Col., *D.S.O.* ... ... ... ... | 10 Miles Road, Clifton. |
| *Bristowe, H. C., M.D. Lond. ... ... ... ... | Wrington, Somerset. |
| *Brown, William, M.D., C.M. Glasg. ... ... | Park View, Fishponds, Bristol. |
| *Buckmaster, G. A., M.A., M.D. Oxon. ... ... | University of Bristol. |
| *Burdon-Cooper, J., M.D., B.Sc. Durh. ... ... | 12 The Circus, Bath. |
| *Bush, J. Paul, *C.M.G.*, *C.B.E.*, Ch.M. Brist. | Vyvyan House, Clifton. |

| | |
|---|---|
| *Cairns, F. J. ... ... ... ... ... ... ... ... | Devon House, Whitehall Road, Bristol. |
| *Carey, R. S., *O.B.E.*, B.A. Cantab. ... ... | 502 Bath Road, Brislington, Bristol. |
| *Carleton, H. H., M.A., M.D. B.Ch. Oxon. | 72 Pembroke Road, Clifton. |
| *Carling, A., M.A., M.B., B.C. Cantab. ... ... | 12 Great George Street, Bristol. |
| *Carwardine, Thomas, M.B., M.S. Lond. ... | 16 Victoria Square, Clifton. |
| *Cave, Edward J., M.D. Lond. ... ... ... ... | 20 The Circus, Bath. |
| *Chambers, E. R. ... ... ... ... ... ... | 9 Clifton Park, Clifton. |
| *Charles, J. R., M.A., M.D., B.C. Cantab. ... | 5 Rodney Place, Clifton. |
| *Chitty, H., M.S. Lond. ... ... ... ... ... | 46 Pembroke Road, Clifton. |
| *Christofferson, P. E., M.B., Ch.B. Brist. ... | 8 Lansdown Place, Clifton. |
| *Clarkmont, L. E. ... ... ... ... ... ... | 5 Rodney Place, Clifton. |
| *Clarke, Cecil, M.B., B.S. Lond. ... ... ... | 3 Fosseway, Clifton. |
| *Clarke, R. C., *O.B.E.*, M.B., Ch.B. Brist. ... | 29 Victoria Square, Clifton. |
| *Coates, Vincent M., *M.C.*, M.A., M.D B.C. Cantab. .. ... ... ... ... ... ... | 10 The Circus, Bath. |
| Connellan, P. S. ... ... ... ... ... ... ... | 12 Bloomsbury Street, London, W.C.1 |
| *Coombs, Carey, M.D. Lond. ... ... ... ... | 3 Pembroke Road, Clifton. |
| *Cooper, William Russell ... ... ... ... | 13 Westfield Park, Redland, Bristol. |
| *Corfield, C., M.D. Durh. ... ... ... ... | 5 Kensington Place, Clifton. |
| Cossham, W. R., M.D., C.M. Aberd.... ... ... | Wellesley House, Cirencester. |
| Cridland, A. B. ... ... ... ... ... ... ... | Salisbury House, Chapel Ash, Wolverhampton. |
| *Cross, F. R., M.B. Lond., LL.D. Brist. ... | Worcester House, Clifton. |
| *Crossman, F. W., M.B. Durh. ... ... ... ... | White's Hill, Hambrook near Bristol. |
| *Crouch, C. Percival, M.B. Lond. ... ... ... | "Penquarry," Weston-super-Mare. |
| *Cummins, Col. S. Lyle, *C.B.*, *C.M.G.*, M.D., N.U.I. ... ... ... ... ... ... ... | Welsh National Memorial Assoc., Westgate Street, Cardiff |

| | |
|---|---|
| *DACRE, JOHN ... ... ... ... ... ... ... ... | 14 Eaton Crescent, Clifton. |
| *DAVIES, E. D. D. ... ... ... ... ... ... ... ... | 6 Lansdown Place, Clifton. |
| *DAVIES, D. S., M.D. Lond., D.P.H. Cantab. ... | 6 Lansdown Place, Clifton. |
| *DAVIS, O. C. M., D.Sc., M.D. Brist. ... ... ... | 90 Cotham Brow, Bristol. |
| *DAWE, J. H., M.B. Edin. ... ... ... ... ... ... | 2 Berkeley Crescent, Clifton. |
| DENING, EDWIN ... ... ... ... ... ... ... ... | Manor House, Stow-on-the-Wold, Glos. |
| DEVINE, H., M.D Lond. ... ... ... ... ... | Corporation Mental Hospital, Portsmouth. |
| *DEVIS, H. F. ... ... ... ... ... ... ... ... ... | 160 Wells Road, Bristol. |
| *DIXON, T. B. ... ... ... ... ... ... ... ... | 134 Coronation Road, Bristol. |
| *DRAPES, T. L., M.B., B.C., B.A. Cantab. ... | St. Maur, Beaufort Square, Chepstow. |
| *DUNBAR, ELIZA L. WALKER, M.D. Zürich ... | 9 Oakfield Road, Clifton. |
| DYMOKE, F. ... ... ... ... ... ... ... ... | 21 Cotham Road, Bristol. |

| | |
|---|---|
| *EDGEWORTH, F. H., M.D., B.C., M.A. Cantab., D.Sc. Lond. ... ... ... ... ... ... ... ... | 13 Richmond Hill, Clifton. |
| EDWARDS, C. D., B.A., M.D. Cantab. ... ... | The Corderies, Chalford, Glos. |
| *ELKINGTON, G. W. ... ... ... ... .. .. ... | Osborne Hotel, Pembroke Road, Clifton. |
| *ELLIOT BROS. LTD. ... ... ... ... ... ... | *Australian Pharmaceutical Notes & News*, O'Connell Street, Sydney, N.S.W. |
| ELLIOTT, F. PERCY, M.B., C.M. Aberd. ... ... | 113 Grove Rd., Walthamstow, London, E.17. |
| *ELLIOTT, W. H. A., M.B., B.S. Lond. ... ... | Tudor Lodge, Cotham Brow, Bristol. |
| *EVERY-CLAYTON, L. E. V., M.D. Lond ... ... | The Old School House, Tetbury. |

| | |
|---|---|
| *FAILL, C. J. C. ... ... ... ... ... ... ... ... | 19 Portland Square, Bristol. |
| FARNFIELD, W. W. ... ... ... .. .. ... ... | Clive Vale, Gillingham, Dorset. |
| *FALLON, Col. J. ... ... ... ... ... ... ... | 35 Cavendish Road, Henleaze, Bristol. |
| *FAWN, G. F. ... ... ... ... ... ... ... ... ... | 6 Oakfield Road, Clifton. |
| *FELLS, A., M.B., C.M. Edin. ... ... ... ... | 6 Elton Road, Clifton. |
| *FIRTH, J. L., M.D., M.S. Lond. ... ... ... ... | 8 Victoria Square, Clifton. |
| *FLEMMING, A. L., M.B., Ch.B. Brist. ... ... | 48 Pembroke Road, Clifton. |
| *FLEMMING C. E. S. ... ... ... ... ... ... | Manvers House, Bradford-on-Avon. |
| *FOSS, E. V., M.D., Ch.B. Brist. ... ... ... | Clouds Hill House, St. George, Bristol. |
| *FRASER, A. D., M.A., M.B., Ch.B. ... ... ... | 60 North View, Westbury Park, Bristol. |
| *FRASER, FORBES, *C.B.E.* ... ... ... ... ... | 5 The Circus, Bath. |

| | |
|---|---|
| *GARDEN, R. R., M.A., M.B., B.Ch. ... ... ... | Guildhall, Bristol. |
| GARLAND, E. B., M.B., C.M. Edin. ... ... ... | 114*a* Hampton Road, Redland, Bristol. |
| GEE, C. A. H., M.B., Ch.B. Brist. ... ... ... | Evercreech, Somerset. |
| *GERRISH, D. S. ... ... ... ... ... ... ... | 328 & 330 Church Road, St. George, Bristol. |
| *GIBBS, A. N. GODBY ... ... ... ... ... ... | 52 Whiteladies Road, Clifton. |
| *GLENNY, E. T., M.B., B.S. Lond. ... ... ... | 102 Pembroke Road, Clifton. |
| *GORDON, R. G. M.D., B.Sc. Edin. ... ... ... | 6 Queen Square, Bath. |
| *GREEN, T. A., *D.S.O.*, M.D., C.M. Edin. ... | 33*a* Whiteladies Road, Clifton. |
| GRIFFITHS, CORNELIUS A. ... ... ... ... ... | 35 Newport Road, Cardiff. |
| *GRIFFITHS, J. S. ... ... .. ... ... ... ... | 20 Redland Park, Bristol. |
| *GROVES, E. W. HEY., M.D., M.S. Lond.... ... | 25 Victoria Square, Clifton. |

| | |
|---|---|
| *HADFIELD, G., M.D. Lond. ... ... ... ... | General Hospital, Bristol. |
| *HAILES CLEMENTS D. G., M.D., C.M. Ed. ... | 2 Richmond Park Road, Clifton. |
| *HALL, E. GEORGE, M.B. Lond. ... ... ... ... | 42 Apsley Road, Clifton, Bristol. |
| *HALL, I. WALKER, M.D., Ch.B.Vict. ... ... | Manilla Lodge, Clifton. |
| *HARRIS, H. ELWIN, B.A., M.B. Cantab. ... ... | 13 Lansdown Place, Clifton. |
| *HARSANT, W. H. ... ... ... ... ... ... ... | Tower House, Clifton Down Road, Clifton. |
| *HARTY, J. P. I., M.B., B.Ch., B.A.O., R.U.I. | West View, Clifton Down, Clifton. |
| *HAWKINS, E. J. ... ... ... ... ... ... ... | Constantine House, Avonmouth. |
| *HERAPATH, C. E. K., *M.C.*, M.D. Lond. ... ... | Ormlie, Clifton. |
| *HERAPATH, C. K. C. ... ... ... ... ... ... | Stoneleigh, Cotham Park, Bristol. |
| *HEREFORD, C. F. A. ... ... ... ... ... ... | 12D Cotham Road, Bristol. |
| *HILL, HEDLEY, M.D. Durh. ... ... ... ... | 101 Pembroke Road, Clifton. |
| *HOLLAND, W. A. L. ... ... ... ... ... ... | Pensions Hospital, Bath. |

HOSPITAL, BRISTOL GENERAL (*Secretary*, T. W. GREGG).

| | |
|---|---|
| *ILES, A. E., *O.B.E.*, M.B., B.S. Lond. ... ... | 17 Victoria Square, Clifton. |

INFIRMARY, BRISTOL ROYAL (*Secretary*, E. C. SMITH).

| | |
|---|---|
| INGLE, C. DURBAN ... ... ... ... ... ... ... | Somerton, Somerset. |

| | |
|---|---|
| *JACKMAN, W A. ... ... ... ... ... ... ... | 24 College Road, Clifton. |
| *JOHNSON, R. G., M.B., D.P.H. ... ... ... ... | 119 Redland Road, Bristol. |
| *JOLL, C. A., M.B., M.S. Lond. ... ... ... ... | 23 New Cavendish Street, W.1. |
| *JONES, J. ELLINGTON ... ... ... ... .. ... | 20 Pembroke Road, Clifton. |

| | |
|---|---|
| *KENNEDY, W. P. M.D. Dubl. ... ... ... ... | 3 Gay Street, Bath. |
| *KERFOOT, STANLEY J. ... ... ... ... ... ... | 109 East Street, Bedminster, Bristol. |
| *KINGSTON, S. H., M.B., Ch.B. Brist. .. ... | 3 Whiteladies Road, Clifton. |
| *KYLE, H. G., M.A., M.D. B.Ch. Oxon. ... ... | 31 Westbury Road, Bristol. |

| | |
|---|---|
| *LACE, F. ... ... ... ... ... ... ... ... ... ... | 5 Gay Street, Bath. |
| *LANSDOWN, R. G. P., M.D., B.S. Durh. ... ... | 39 Oakfield Road, Clifton. |
| *LAVINGTON, C. C., M.B., B.S. Durh. ... ... | 1 Burlington Road, Redland, Bristol. |
| *LEES, E. LEONARD, M.D., C.M. Ed. ... ... ... | 1 Chesterfield Place, Clifton. |
| *LEGAT, R. EDDOWES M.B., C.M. Edin.... ... | Murray House Staple Hill, Bristol. |

### LIST OF SUBSCRIBERS.

| | |
|---|---|
| LEIGH, W. W. ... ... ... ... ... ... ... ... | Treharris, Glamorganshire. |
| *LEONARD, R. C., M.D. Durham ... ... ... ... | Bower Ashton, Bristol. |
| *LEVIS, J. S., *M.C.*, M.B., B.Ch., N.U.I. ... | 20 Gay Street, Bath. |
| *LEWIS, D. J. ... ... ... ... ... ... ... ... | 11 Gt. Bedford Street, Bath. |
| *LINTON, MARION S., M.B. Lond. ... ... ... | 21 Oakfield Road, Clifton. |
| *LISTER, L. MARGARET ... ... ... ... ... | 12 All Saints Road, Clifton. |
| *LOGAN, H. B. ... ... ... ... ... ... ... ... | Eastfield, Southville, Bristol. |
| *LUCAS, J. J. S., M.D. Lond. ... ... ... ... ... | 186 Wells Road, Totterdown, Bristol. |

| | |
|---|---|
| MACKINNON, CHARLES, M.B., C.M., M.A. Glasg. | Davaar, Cirencester. |
| MACLEAN, EWEN J., M.D., C.M. Ed.... ... ... | 12 Park Place, Cardiff. |
| MACLEAN, J. CAMPBELL, M.B., C.M. Ed.... ... | Swindon House, Swindon. |
| *MACLEOD, DONALD, M.B., C.M. Ed. ... ... | 84 Redland Road, Redland, Bristol. |
| *MACLEOD, G., *M.C.*, M.D., Ch.B. ... ... ... | "Wargrave," Clevedon. |
| *MACWATTERS, J. C., *M.B.E.* ... ... ... ... | Almondsbury, near Bristol. |
| *MCKENZIE, W. G., *M.C.* ... ... ... ... ... | 101 Coronation Road, Bristol. |
| *MCLANNAHAN, J. G. ... ... ... ... .. ... | The Mount, Stonehouse. |
| MARSHALL, ARTHUR L., M.B., B.A. Cantab. ... | Thornleigh, Upper Clapton, London, E.5 |
| *MATHER, J. S., M.B., C.M. Aberd. ... ... ... | Lawrence Hill House, Bristol. |
| *MAYES, F. J. A. ... ... ... ... ... ... ... | 79 Pembroke Road, Clifton. |
| MIALL, C. L'O. ... ... ... ... ... ... ... | Elm Hayes, Paulton, near Bristol. |
| *MOORE, C. A., M.B., M.S. Lond.... ... ... ... | 56 St. Paul's Road, Clifton. |
| *MOORE, L. A., M.B., Ch.B. Brist. ... ... ... | 102 Redland Road, Bristol. |
| *MORRIS, A. G. ... ... ... ... ... ... ... | 18 Westbury Park, Westbury-on-Trym. |
| MORRIS, L. N., M.B., Ch.B. Brist. ... ... ... | 24 All Hallows Road, Upper Easton, near Bristol. |
| *MORRIS, MARY E. H., M.D. Lond. ... ... ... | 19 Gay Street, Bath. |
| *MORTON, C. A. ... ... ... ... ... ... ... ... | 14 Vyvyan Terrace, Clifton. |
| *MUMFORD, W. G., M.B. Lond.... ... ... ... | 18 The Circus, Bath. |
| *MYLES, G T. ... ... ... ... ... ... ... ... | 7 Apsley Road, Clifton. |

| | |
|---|---|
| *NEILD, NEWMAN, M.B., B.Ch. Vict. ... ... ... | 9 Richmond Hill, Clifton. |
| *NEWNHAM, W. H. C., M.B., M.A. Cantab. ... | 3 Lansdown Place, Victoria Square, Clifton. |
| *NICHOLS, F. C., *M.C.*, M.B., Ch.B. Brist. ... | 38 Whiteladies Road, Clifton. |
| *NIXON, J. A., *C.M.G.*, M.D. Cantab. ... ... | 7 Lansdown Place, Clifton. |
| *NORGATE, R. H. .. ... ... .. ... ... ... | Southmead Infirmary, Bristol. |

| | |
|---|---|
| *O'KEEFFE, D. S., M.B., B.Ch., R.U.I. ... ... | 12 Elton Road, Clifton. |
| OPPENHEIMER, SON & CO. LTD. ... ... ... | 179 Queen Victoria Street, London, E.C.4. |
| *ORMEROD, H. LAWRENCE, M.B., B.Ch. R.U.I. | 2 Henleaze Road, Westbury Park, Bristol. |
| *ORR-EWING, H. J., *M.C.*, M.D. Lond. ... ... | English Mission Hospital, Jerusalem. |

| | |
|---|---|
| *PARKER, GEORGE, M.D., M.A. Cantab. ... ... | 14 Pembroke Road, Clifton. |
| *PARKES, J. A., M.B., Ch.B. Liverpool. ... ... | 2 Effingham Road, Bristol. |
| PARKINSON, Major G. S., *D.S.O.*, R.A.M.C.... | M.O.H., Gibraltar. |

### LIST OF SUBSCRIBERS.

| | |
|---|---|
| *Parsons, H. F. ... ... ... ... ... ... ... ... | The Heath, Hazelwood Road, Sneyd Park, |
| Parsons, Sir J. H., M.B., B.S., D.Sc. Lond... | 54 Queen Anne Street, London, W. |
| Paterson, Donald Rose, M.D., C.M. Ed. ... | 15 St. Andrew's Crescent, Cardiff. |
| *Pickering, C. F. ... ... ... ... ... ... ... ... | 13 Berkeley Square, Bristol. |
| *Pollard, J., M.B., B.Ch., B.A.O. ... ... ... | 52 Cumberland Road, Bristol. |
| Powell, J. J., M.D. Lond. ... ... ... ... ... ... | 2 Gloucester Road, Bishopston, Bristol. |
| Price, D. T. M.B. Lond. ... ... ... ... ... ... | Castle Cary, Somerset. |
| *Prichard, Arthur W. ... ... ... ... ... ... ... | 6 Rodney Place, Clifton. |
| *Pridie, H. H., M.B., C.M. Ed. ... ... ... | 17 Oakfield Road, Clifton. |
| *Prowse, Arthur B., M.D. Lond. ... ... ... | 5 Lansdown Place, Clifton. |

| | |
|---|---|
| *Rayner, D. C. ... ... ... ... ... ... ... ... | 9 Lansdown Place, Clifton, Bristol. |
| *Renton, R. A. S., *M.C.*, M.D. Durh.... ... ... | Tortworth House, Clevedon. |
| Ritchie, Thomas P. ... ... ... ... ... ... ... | 27 Oakfield Road, Clifton. |
| *Robertson, D., M.B. Ch.B. Edin. ... ... ... | 205 Wells Road, Knowle. |
| *Rogers, Beatrice, ... ... ... ... .. .. ... | Tower House, Whiteladies Road, Clifton. |
| *Rogers, B. M. H., M.D., B.Ch., B.A. Oxon. ... | 14 Mortimer Road, Clifton. |
| *Rolfe, R., B.A., M.B., B.C. Cantab. ... ... | Park House, St. Andrews Road, Avonmouth. |
| *Rose, F. H. ... ... ... ... ... ... ... ... ... | 8 Chantry Road, Clifton. |
| *Rudge, C. King ... ... ... ... ... ... ... .. | 145 Whiteladies Road Bristol. |
| *Rutherford, J. M., M.B., C.M. Edin. ... ... | Brislington House, near Bristol. |

| | |
|---|---|
| *Salisbury, Walter, M.D., M.S. Lond. ... | 4 Durdham Park, Clifton. |
| *Shaw J. E., M.B., C.M., Edinb. ... ... ... | 23 Caledonia Place Clifton. |
| *Short, A. R., M.D., B.S., B.Sc. Lond. ... ... | 69 Pembroke Road, Clifton. |
| *Short, L. J., M.D., B.S. Lond., M.D. Brist. ... | Grove Park House, Weston-super-Mare. |
| *Smith, D. Munro ... ... ... ... ... ... ... ... | 479 Fishponds Road, Bristol. |
| *Smith, G. Cockburn, M.D. Brux. ... ... ... | 12 South Road, Newton Abbot. |
| Smith, Rev. Reginald, M.A. Cantab. ... ... | The Vicarage, Walpole St. Andrew, Norfolk. |
| *Smith, W. Alexander, M.B., M.A. Oxon. ... | 70 Pembroke Road, Clifton. |
| *Smyth, Richard, M.A., M.D. Dub. ... ... | Burnley, Westbury-on-Trym. |
| Society, Cardiff Medical (*Hon. Lib.*, Queen Street, Cardiff). | |
| *Stock, W. S. V., M.B., B.S. Lond. ... ... ... | 1 Mortimer Road, Clifton. |
| *Statham, R. S. S., *O.B.E.*, M.D., M.Ch. Brist. | 24 College Road, Clifton. |
| *Swain, James, *C.B.*, *C.B.E.*, M.D., M.S. Lond. | 4 Victoria Square, Clifton. |
| *Swayne, W. Carless, M.D. Lond. ... ... ... | 2 Clifton Park, Clifton. |
| *Symes, J. O., M.D. Lond. ... ... ... ... ... | 71 Pembroke Road, Clifton. |
| *Smythe, H. J. D., *M.C.*, M.B., M.S. ... ... | 72 St. Paul's, Road, Clifton. |

### LIST OF SUBSCRIBERS.

| | |
|---|---|
| *Tasker, D. G. C., M.B., M.S. Lond. ... ... | Bristol General Hospital. |
| *Tate, Colonel G. W. *C.M.G.*, *D.S.O.*, M.B., B.Ch. Dub. ... ... ... ... ... ... ... | 8 Park Mansions, Park Street, Bath. |
| Taylor, A. L. ... .. ... .. ... ... ... ... | The Royal Infirmary, Bristol. |
| *Taylor, H. N., M.A. Cantab., M.D. Dub. ... | Hillside, Ebbw Vale. |
| *Taylor, James ... ... ... ... ... ... ... ... | 36 Alma Road, Clifton. |
| *Temple, G. H., M.B., C.M. Ed. ... ... ... | Ailanthus, Weston-super-Mare. |
| *Terry, C. H., M.A., M.B., B.Ch. Oxon. .. ... | 2 Gay Street, Bath. |
| Thin, James ... ... ... ... ... ... ... ... ... | 54 & 55 South Bridge, Edinburgh. |
| *Thomas, J. D., M.B. Cantab. ... ... ... ... | Northwoods, Winterbourne. |
| *Todd, A. T., *O.B.E.*, M.B., Ch.B. Edin. ... | Clayton, Clifton Park, Clifton. |
| *Trist, J. R. R., *M.C.* ... ... ... ... ... ... | Ministry of Pensions, Clifton Down, Clifton. |
| *Vickery, W. H. ... ... ... ... ... ... ... | 1 Trewartha Park, Weston-super-Mare. |
| *Walker, Cyril H., M.B., B.A. Cantab. ... ... | 8 Oakfield Road, Clifton. |
| *Wallace, John, *O.B.E.*, M.B., C.M. Edin. ... | Lauriston, Weston-super-Mare. |
| *Wallace, J. T., M.B., B.Ch., B.A. R.U.I. ... | 48 Coronation Road, Bristol. |
| *Walters, C. F. ... ... ... ... .. ... ... | 5 Mortimer Road, Clifton. |
| *Wanklyn-James, C. W., *O.B.E.* ... ... ... | 3 Mortimer Road, Clifton. |
| Warren, R., M.A., M.D. ... ... ... ... ... | 1 Royal Terrace, Weston-super-Mare. |
| *Waterhouse, R., M.D. Lond. .. ... ... ... | 25 Circus, Bath. |
| *Watson-Williams, P., M.D. Lond.... ... ... | 2 Rodney Place, Clifton. |
| *Watson-Williams, E., *M.C.*, M.B., B.Ch., B.A. Cantab. ... ... ... ... ... ... ... | 12 Victoria Square, Clifton. |
| *Whicher, A. H. ... ... ... ... ... ... ... | 115 Queen's Road, Clifton. |
| Whitehouse, H. Beckwith, M.S. ... ... | 52 Newhall Street, Birmingham. |
| Wigan, C. A., M.D. Durh., J.P. ... ... ... ... | Deepdene, Portishead. |
| Wigmore, J., M.D. R. U. I. ... ... ... ... ... | 16 Queen Square, Bath. |
| *Wilkinson, S. H., M.D. Edin. ... ... ... | Pensions Hospital, Ashton Court. |
| *Willcox, J. W. J., M.B., B.S. Lond. ... ... | Glastonbury. |
| Willcox, R. Lewis ... ... ... ... ... ... | 97 London Road, Salisbury. |
| *Williams, E. C., M.B., B.C., B.A. Cantab. ... | 159 Whiteladies Road, Clifton. |
| *Williams, G. C., B.A. Cantab. ... ... ... | Ham Green, Pill. |
| Williams, H. Egerton ... ... ... ... ... | The Mount, Newport, Mon. |
| *Williams, L. H., M.D. Durh. ... ... ... ... | Thornbury, Glos. |
| Williams, S. R., M.B. Lond. ... ... ... ... | Buckfastleigh, Devon. |
| *Wills, W. K., M.B., B.C. Cantab. ... ... ... | 19 Whiteladies Road, Clifton. |
| *Windsor-Aubrey, H. W. ... ... ... ... ... | 79 Coronation Road, Bristol. |
| *Wood, Duncan ... ... ... ... ... ... ... | 5 Eaton Crescent, Clifton. |
| *Wood, W. V., *M.C.* ... ... ... ... ... ... | Henley Lodge, Yatton, Som. |
| *Woodcock, O. H., M.D., Ch.B. Man. ... ... | Dunmurray, Sneyd Park, Clifton. |
| *Wright, A. J., M.B., B.S. Lond. ... ... ... | 14 Victoria Square, Clifton. |

UNIVERSITY OF BRISTOL.—Students of the University have recently passed the following examinations :—

M.B., B.S. LOND.—R. B. Britton.

F.R.C.S.—*Primary Examination :* S. P. Taylor.

L.D.S., R.C.S.—*Chemistry and Physics :* G. Hazell. *Dental Mechanics :* C. Morgan, J. W. Cooper, G. Meadows. *Dental Metallurgy :* G. Meadows, G. S. Williams. *Anatomy and Physiology :* G. S. Williams, K. Huxtable, C. E. Down, J. W. Cooper, G. Meadows, B. A. Butcher, N. H. Simmonds. *Dental Anatomy and Physiology :* G. Meadows. *2nd Examination, Part I. :* D. J. Andrews, S. G. House. *2nd Examination, Part II. :* *A. J. Constance, *W. G. Smith, *H. H. Watkins.

* Denotes Qualification.

### Bristol Medico-Chirurgical Society.

### President.

J. A. NIXON, C.M.G., M.D. Cantab.

### President-Elect.

J. LACY FIRTH, M.D., M.S. Lond.

### Committee.

*Ex-Officio*

- C. H. WALKER, M.B., B.A. Cantab. (Retiring President).

- P. WATSON-WILLIAMS, M.D. Lond. (Editor of *Journal*).

- C K. RUDGE (Hon. Librarian).

- A. L. FLEMMING, M.B., Ch.B. Brist. (Editorial Secretary).

*Elected.*

F. LACE
R. G. P. LANSDOWN, M.D., B.S. Durh. } Ex-Presidents.
J. LACY FIRTH, M.D., M.S. Lond.
J. O. SYMES, M.D. Lond.
T. CARWARDINE, M.S. Lond.
D. C. RAYNER.
FORBES FRASER.
C. FERRIER WALTERS.

### Honorary Secretary.

S. V. STOCK, M.B., B.S. Lond.

Medical Practitioners wishing to join the Society are requested to communicate with the Hon. Secretary, Mr. S. V. Stock, 1 Mortimer Road, Clifton. The Annual Subscription is £1 11s. 6d.

---

*Extracts from Journal By-laws.*

4.—The *Journal* shall be delivered free to Subscribers and also to Members of the Society whose subscriptions are not in arrear.

6.—The Annual Subscription to the *Journal* is 10/6.

---

Communications intended for insertion in the *Journal* should be sent to the Editor, Dr. P. Watson-Williams, 2 Rodney Place, Clifton, Bristol.

Books for Review and Exchange Journals should be sent to the Assistant-Editor, *Bristol Medico-Chirurgical Journal*, Medical Library, The University, Bristol.

Communications referring to the delivery of the *Journal* should be sent to the Editorial Secretary, A. L. Flemming, 48 Pembroke Road, Clifton, Bristol, to whom Subscriptions are to be paid *in advance*.

Cases for Binding Vols. I—XXXIX are now ready, and may be obtained, price 1/9 each (postage extra), upon application to J. W. Arrowsmith Ltd., Quay Street, Bristol; or the *Journal* will be bound, including Case, for 7/- per volume.

### *PUBLICATIONS RECEIVED.*

---

From Baillière, Tindall and Cox, London :

*Lawson Tait: his Life and Work.* By W. J. Stewart McKay, M.B. 1922. Price 25s. net.

*Practical Anæsthetics.* By Charles F. Hadfield, M.B.E., M.A., M.D. 1923. Price 7s. 6d. net.

From Octave Doin, Paris :

*Greffes testiculaires.* Par le Docteur Serge Voronoff. 1923.

From Wm. Heinemann (Medical Books) Ltd., London :

*Anæsthetics in Practice and Theory.* By J. Blomfield, O.B.E., M.D. 1922. Price 25s. net.

From H. K. Lewis and Co. Ltd., London :

*Practical Handbook on the Diseases of Children.* By Bernard Myers, C.M.G., M.D. 1922. Price 21s. net.

*Ophthalmic Surgery.* By Major V. Nesfield, F.R.C.S. 1922. Price 9s. net.

From Macmillan and Co., Ltd., London :

*Some Medical Aspects of Old Age.* By Sir Humphry Rolleston, K.C.B., M.D., 1922. Price 6s. net.

From John Murray, London :

*Saint Bartholomew's Hospital Reports.* Volume LV. 1922.

From Oxford Medical Publications, London :

*A Textbook of the Practice of Medicine.* By Various Authors. Edited by Frederick W. Price, M.D. [1922.] Price 35s. net.

*A Manual of Surgical Anatomy.* By Lewis Beesly, F.R.C.S.E., and T. B. Johnston M.B., Ch.B. Second Edition. 1922. Price 18s. net.

*Anæsthetics and their Administration.* By the late Sir Frederic W. Hewitt, M.V.O., M.A., M.D. Fifth Edition. Edited by Henry Robinson, M.A., M.D. 1922. Price 30s. net.

*Cunningham's Text-book of Anatomy.* Edited by Arthur Robinson, M.D. Fifth Edition. [1922.] Price £2 2s. net.

From The Temperance Council of the Christian Churches, London :

*The Church and the Drink Evil.* Edited by Henry Carter. [1922.] Price 1s. 6d.

From John Wright and Sons Ltd., Bristol :

*An Index of Prognosis.* By Various Writers. Edited by A. Rendle Short, M.D., B.S., F.R.C.S. Third Edition. 1922. Price £2 2s. net.

*The New Physiology in Surgical and General Practice.* By A. Rendle Short, M.D., B.S., F.R.C.S. Fifth Edition, revised and enlarged. 1922. Price 9s. 6d. net.

### THE Hospital and Health REVIEW

A JOURNAL FOR EVERYONE INTERESTED IN PUBLIC HEALTH, PREVENTIVE AND CURATIVE MEDICINE AND HOSPITAL AND INSTITUTIONAL WORK.

*Established 1886.*

Published MONTHLY, Price 6d.

THE HOSPITAL AND HEALTH REVIEW includes within its sphere the various aspects of PUBLIC AND PERSONAL HEALTH, PREVENTIVE AND CURATIVE MEDICINE, HOSPITAL AND INSTITUTION WORK, AND THE GENERAL WELL-BEING OF THE PEOPLE. These subjects of truly vital concern are dealt with in plain, and as far as possible non-technical, language, so that they may be understood, and consequently of *service to everyone* interested in these matters.

Price 6d. of all Booksellers and Newsagents, or 7½d. post free of the Manager, "The Hospital and Health Review," 28/29 Southampton Street, Strand, London, W.C. 2.

